# Primary systemic therapy for patients with brain metastases from lung cancer ineligible for targeted agents

**DOI:** 10.1007/s00432-022-03919-0

**Published:** 2022-01-12

**Authors:** Carsten Nieder, Siv G. Aanes, Ellinor Haukland

**Affiliations:** 1 Department of Oncology and Palliative Medicine, Nordland Hospital, 8092 Bodø, Norway; 2 Department of Clinical Medicine, Faculty of Health Sciences, UiT—The Arctic University of Norway, 9037 Tromsø, Norway; 3 SHARE—Center for Resilience in Healthcare, Faculty of Health Sciences, Department of Quality and Health Technology, University of Stavanger, 4036 Stavanger, Norway

**Keywords:** Lung cancer, Brain metastases, Chemotherapy, Systemic therapy, Survival, Pattern of care

## Abstract

**Purpose:**

The purpose of this study was to evaluate overall survival after systemic therapy, largely chemotherapy, in patients with small cell or non-small cell lung cancer and brain metastases. After completion of systemic therapy, some patients received planned brain irradiation, while others were followed.

**Methods:**

Retrospective cohort study.

**Results:**

Thirty-eight patients were included (28 small cell, 20 followed with imaging). Six of these 20 patients (30%) received delayed radiotherapy during follow-up. Planned radiotherapy (*n* = 18, intention-to-treat) was associated with longer survival from diagnosis of brain metastases, median 10.8 versus 6.1 months, *p* = 0.025. Delayed radiotherapy still resulted in numerically better survival than no radiotherapy at all (median 8.8 versus 5.3 months, not significant). If calculated from the start of delayed radiotherapy, median survival was only 2.7 months. In a multivariable analysis, both Karnofsky performance status ≥ 70 (*p* = 0.03) and planned radiotherapy (*p* = 0.05) were associated with better survival.

**Conclusion:**

In patients ineligible for targeted agents, planned radiotherapy in a modern treatment setting was associated with longer survival compared to no radiotherapy. Timing and type of radiotherapy in such patients should be evaluated in prospective trials to identify patients who might not need planned radiotherapy.

## Introduction

With increasing primary tumor (T) and lymph-node (N) stage, both small cell lung cancer (SCLC) and non-small cell lung cancer (NSCLC) tend to metastasize to the brain (Sacks and Rahman 2020). Certain molecular features of NSCLC, e.g., epidermal growth factor receptor (EGFR) mutations, modulate the risk of brain metastases, while also representing targets for primary systemic therapy (Andratschke et al. 2019; Wang et al. 2021). A complex decision-making is required when recommending primary systemic therapy. For example, the simultaneous presence of widespread extracranial disease, an actionable mutation and limited, asymptomatic brain disease may prompt the multidisciplinary tumor board (MDT) to recommend this strategy. Despite a long-standing, historical interest in primary cytotoxic chemotherapy for selected patients with SCLC and NSCLC with brain metastases (Minotti et al. 1998; Bernardo et al. 2002; Chen et al. 2008; Lee et al. 2008), early administration of radiotherapy was common (Nieder et al. 2019a, b; Kepka et al. 2021; Du et al. 2021). Under special circumstances, surgical resection of a single brain metastasis may be a viable option (Fuchs et al. 2021).

Also at the authors’ institution, which has an extremely low rate of patients with EGFR mutations or other targetable alterations influencing first-line treatment (Nieder et al. 2020), early radiotherapy was the prevailing strategy for patients with brain metastases from lung cancer undergoing active treatment rather than supportive, palliative measures. Nevertheless, on a case-by-case basis, the MDT sometimes recommended primary cytotoxic chemotherapy and recently immune checkpoint inhibitors (ICI) or ICI plus chemotherapy if no actionable target was present. The purpose of the present study was to evaluate the survival and several secondary outcomes in these consecutively treated patients with SCLC or NSCLC.

## Patients and methods

A retrospective single-institution quality-of-care analysis was performed, based on electronic health records and a previously utilized brain metastases database, which does not include patients with leptomeningeal spread (Nieder et al. 2019b, 2020). Magnetic resonance imaging was used to diagnose the brain metastases, without histological confirmation. After exclusion of patients managed with best supportive care or upfront local treatment (surgery, any type of radiotherapy), those who received primary drug-based treatment for SCLC or NSCLC without actionable mutations in the time period 2007–2020 were included. Consolidation radiotherapy after the last cycle of chemotherapy was permitted. However, sandwich radiotherapy between the initial cycles of chemotherapy (upfront combined treatment) was not permitted. None of the patients had received prophylactic cranial irradiation (PCI). Both synchronous and metachronous brain metastases were permitted. The MDT selected the type of systemic therapy according to national Norwegian guidelines provided by the Norwegian Lung Cancer Group. If indicated, maintenance treatment was administered. Later during the course of the disease, further lines of systemic therapy and delayed (salvage) brain-directed treatments could be administered, as judged appropriate by the MDT. For example, one patient who started with chemotherapy, later received three sequential courses of stereotactic radiosurgery before proceeding to whole-brain irradiation. None of the patients received targeted agents in any line of treatment. Bevacizumab was not utilized.

Actuarial survival from the day of treatment initiation was calculated with the Kaplan–Meier method and compared between subgroups with the log-rank test. Thirty-two patients had died and six patients were censored at the time of their last follow-up (median 18 months). Associations between different variables of interest were assessed with the Chi-square or Fisher’s exact probability test (two-tailed). The validated prognostic score LabBM (serum hemoglobin, platelets, albumin, C-reactive protein, and lactate dehydrogenase) was calculated as previously described (Berghoff et al. 2017; Nieder et al. 2019c). The impact of continuous variables such as age and number of brain metastases on survival was examined in univariate Cox analyses. A multivariate forward conditional Cox analysis of prognostic factors for survival was then performed. A *p* value < 0.05 was considered statistically significant. Due to limited patient numbers, a *p* value < 0.15 was selected in the initial analyses, e.g., when deciding which parameters to include in the multivariate Cox model. Intra- and extracranial response rates and patterns of progression were not evaluated. The database created for the purpose of this quality-of-care analysis does not require approval by the local Ethics Committee (REK Nord).

## Results

**Fig. 1:**
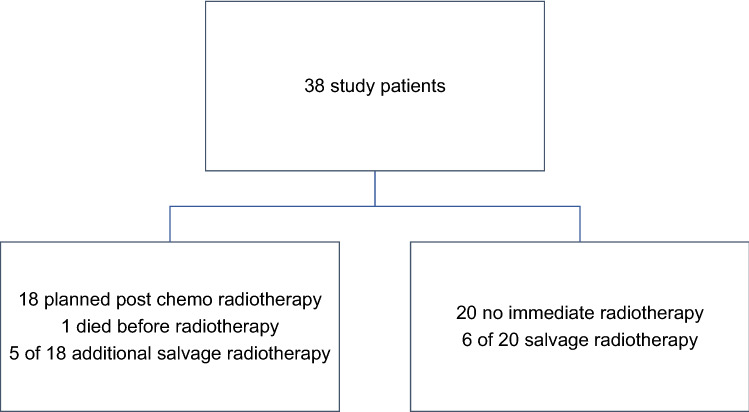
Study diagram

Among 38 patients included in the study, 28 had SCLC (74%), seven adenocarcinoma (18%), two neuroendocrine NSCLC, and one squamous cell carcinoma. Except for two patients, all had their brain metastases diagnosed when the initial diagnosis of lung cancer was made (synchronous brain metastases). In ten patients (26%), clinical symptoms of brain metastases were present. The others had staging-detected, asymptomatic lesions. Further baseline data are shown in Table 1. Systemic therapy consisted of platinum-based doublets in 32 patients (84%), typically four cycles. Six patients with NSCLC received ICI monotherapy (*n* = 2) or ICI with platinum doublet chemotherapy (*n* = 4). As indicated in Fig. 1, 18 patients (47%) were scheduled for planned radiotherapy after their first line of systemic treatment, while 20 patients (53%) were followed with brain imaging. Age, Karnofsky performance status (KPS), presence of symptoms, and number of lesions were not significantly different between the two groups. However, significantly more patients without extracranial metastases were scheduled for planned radiotherapy (80 versus 36%, *p* = 0.03). Six of 20 patients (30%) followed with imaging surveillance received delayed or salvage radiotherapy during the course of the disease. Among patients with planned radiotherapy, 5 (28%) received at least one more course of brain irradiation due to progression or development of new lesions (Table 2).

**Table 1:** Patient characteristics (*n* = 38, time period 2007–2020)

Parameter	*n*	%
Female sex	20	53
Male sex	18	47
No extracranial metastases	10	26
Liver metastases	9	24
Bone metastases	12	32
Only one extracranial distant site affected	7	18
Two such organs affected, e.g., bone + liver	13	34
Three or more organs affected	8	21
No brain radiotherapy at all	15	39
Karnofsky performance status 90–100	7	18
Karnofsky performance status 70–80	27	71
Karnofsky performance status < 70	4	11
Whole-brain radiotherapy*	19	50
Higher dose radiotherapy	4	11
Single brain metastasis	10	26
Median number of brain metastases, range	3	1–50
Median size of the largest lesion, range (mm)	10	3–30
Median age, range (years)	68.5	46–82
Median LabBM score, range (points)	1.5	0–3

*18 patients had received 30 Gy in 10 fractions of 3 Gy and one 25 Gy in 10 fractions of 2.5 Gy (higher dose means stereotactic radiotherapy without whole-brain radiotherapy (*n* = 2) or simultaneous integrated boost whole-brain radiotherapy (*n* = 2))

**Table 2:** Detailed description of re-irradiated patients (*n* = 5)

Sex, age (years)	Tumor characteristics	Treatment	First radiotherapy	Second radiotherapy	Survival*
Male, 67	SCLC, asymptomatic single BM, skin and soft tissue metastases (Dec. 2015)	Planned WBRT	WBRT 10 fractions of 3 Gy (Mar. 2016)	SRS (Jan. 2017)	19.0
Female, 69	SCLC, 8 asymptomatic BM, bone and pleural metastases (Jun. 2018)	Planned WBRT	WBRT 10 fractions of 3 Gy (Sep. 2018)	WBRT 10 fractions of 2.5 Gy (plus 0.5 Gy SIB; Oct. 2019)	19.0
Female, 69	SCLC, 3 asymptomatic BM, adrenal gland metastases (Aug. 2019)	Planned WBRT	WBRT 10 fractions of 3 Gy (plus 0.5 Gy SIB; Dec. 2019)	SFRT 6 fractions of 5 Gy (Aug. 2020)	19.2
Female, 76	Squamous cell cancer, 3 asymptomatic BM, no extracranial met. (May 2019)	Planned SRS	SRS (Sep. 2019)	SRS (Dec. 2019 and Feb. 2020), WBRT 10 fractions of 3 Gy (Aug. 2020)	25.2 +
Female, 61	SCLC, 8 asymptomatic BM, adrenal gland and lung met. (Aug. 2019)	Planned WBRT	WBRT 10 fractions of 3 Gy (Dec. 2019)	SFRT 7 fractions of 4.5 Gy (Jul. 2020)	15.4

*SCLC* small cell lung cancer, *BM* brain metastases, *CTx* platinum-based chemotherapy, *WBRT* whole-brain radiotherapy, *SIB* simultaneous integrated boost, *SRS* stereotactic radiosurgery, *SFRT* stereotactic fractionated radiotherapy

*Months from diagnosis of brain metastases (+ ongoing)

**Fig. 2:**
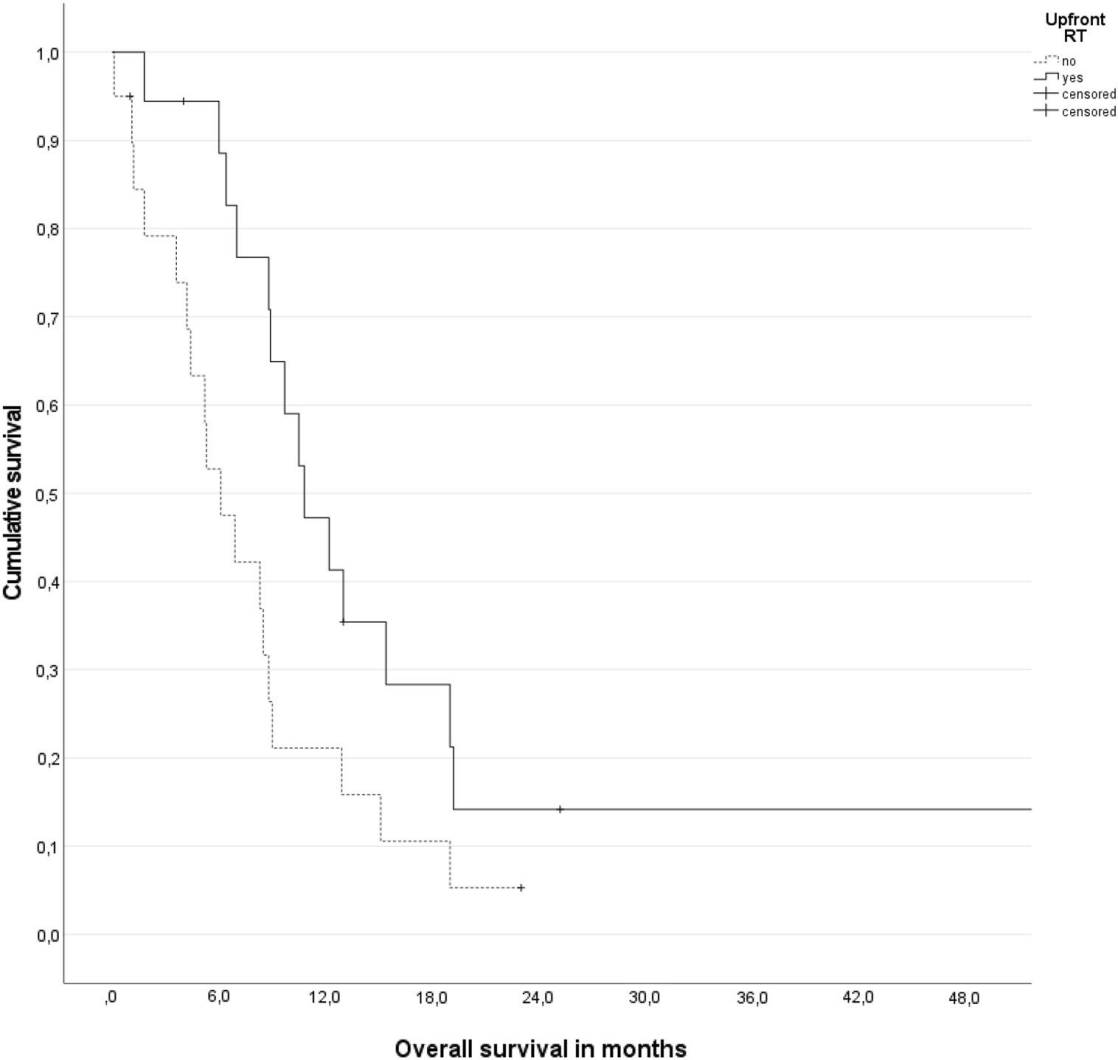
Actuarial overall survival (Kaplan–Meier plot) stratified for planned radiotherapy (intention-to-treat, *n* = 18), *p* = 0.025 (log-rank test)

For all patients, median overall survival was 8.8 months (95% confidence interval (CI) 7.9–9.7 months). For all irradiated patients regardless of timing, median overall survival from the start of radiotherapy was 7.1 months (95% CI 4.2–10.0 months). Planned radiotherapy (*n* = 18 on an intention-to-treat basis) was associated with longer survival from diagnosis of brain metastases, median 10.8 versus 6.1 months, *p* = 0.025 (Fig. 2). Delayed salvage radiotherapy still resulted in numerically better survival than no radiotherapy at all (median 8.8 versus 5.3 months, not significant). If calculated from the start of delayed salvage radiotherapy, median survival was only 2.7 months.

Regarding survival after diagnosis of brain metastases, all baseline parameters summarized in the Results section including Table 1 were tested for their prognostic impact. Several prognostic factors known from previous studies of radiotherapy were not associated with this endpoint (age, number, and size of brain metastases, LabBM score; all *p* > 0.15). The same was true for the diagnostic setting (symptoms yes/no; *p* = 0.22) and histologic type of lung cancer (*p* = 0.3). In contrast, the absence of extracranial metastases was potentially important (median survival 10.5 versus 8.3 months, *p* = 0.06). Reduced survival was observed in the presence of liver metastases (5.2 versus 9.7 months, *p* = 0.05) and bone metastases (6.1 versus 9.0 months, *p* = 0.08). KPS < 70 was highly significant (median 4.2 versus 8.9 months, *p* = 0.005). These four parameters and the radiotherapy setting (planned versus imaging follow-up/surveillance) were included in the multivariable Cox model. Both KPS ≥ 70 (*p* = 0.03) and planned radiotherapy (*p* = 0.05) were more important than absence of extracranial metastases (*p* = 0.18), no liver metastases (*p* = 0.28), and no bone metastases (*p* = 0.45).

Among 32 patients who had died, the cause of death was unknown in three, intracranial progression in five, extracranial progression in 17, and difficult to judge in seven who had active brain metastases, but also extracranial progression at their last hospital visit and who died at home or in a nursing home.

## Discussion

As shown in older studies (Minotti et al. 1998; Bernardo et al. 2002; Chen et al. 2008; Lee et al. 2008), the fact that brain metastases with permeable blood–brain barrier may respond to systemic chemotherapy has stimulated researchers to pursue strategies of delayed or completely eliminated brain irradiation, particularly after the advent of targeted agents for molecularly defined types of NSCLC and in the era of effective ICI therapy. Most patients harbor both, brain and extracranial distant metastases (Nieder et al. 2021a), are not in an oligometastatic setting and require systemic treatment to optimize survival outcomes (Nieder et al. 2021b). It is not always easy to select the patients who are likely to derive added value from brain irradiation, a treatment which also can add toxicity. The present study evaluated patients with brain metastases from lung cancer not eligible for targeted drugs such as EGFR tyrosine kinase inhibitors, who initially received systemic therapy, the approach recommended by the institutional MDT for selected patients. Because this strategy still causes debate, our group was interested in the patterns of care and outcomes.

The retrospective study included patients mainly with SCLC, synchronous brain metastases, often but not always asymptomatic lesions, and highly variable extent of brain involvement. In other words, a relatively heterogeneous population was examined, also with regard to treatment. Typically, platinum-based doublets were prescribed and, in case of radiotherapy, whole-brain irradiation (WBRT). Significantly, more patients without extracranial metastases were scheduled for planned radiotherapy (80 versus 36%, *p* = 0.03), a strategy pursued in almost half of the cohort. Six of 20 patients (30%) followed with imaging surveillance received delayed or salvage radiotherapy during the course of the disease. This finding supports those of the older studies, which already had suggested that not all patients require brain irradiation. Among patients with planned radiotherapy, 5 (28%) received at least one more course of brain irradiation due to progression or development of new lesions. In other words, planned radiotherapy does not completely eliminate the threat of brain progression and need for additional treatment. Ideally, clinicians would be able to perform better patient selection at diagnosis, resulting in less need for sequential salvage radiotherapy. On the other hand, in the era of reduced WBRT utilization, sequential administration of several courses of focal radiotherapy has become increasingly accepted (Nieder et al. 2018a).

We found that patients scheduled for planned radiotherapy after completion of systemic treatment had the longest survival. Delayed salvage radiotherapy resulted in a median survival of only 2.7 months. Selection bias often explains why a certain treatment strategy results in superior outcome in non-randomized studies like the present one. The main imbalance between the groups with and without planned radiotherapy was the presence or absence of extracranial metastases. However, after accounting for this and other baseline parameters, the multivariable regression analysis still suggested that planned radiotherapy may improve survival. A definitive recommendation would require additional evidence and data on important aspects of survivorship such as quality of life, cognitive, and neurologic outcomes. The latter require prospective data collection. In the present study, patient-reported outcomes were not available. Given that extracranial progression was a common cause of death, further improvement of extracranial disease control and systemic treatment is needed. In this context, the progress made for NSCLC was more remarkable than for SCLC, the predominant type of cancer in our study. In SCLC, PCI has been shown to improve survival (Kepka et al. 2021). One might argue that the common setting of four cycles of chemotherapy followed by planned WBRT in many SCLC patients in our study resembles the PCI concept. Overall, the median survival in this study (8.8 months) was limited, and so was long-term survival. Previously, we reported a median survival of 5.4 months in patients who received radiotherapy for brain metastases from lung cancer (Nieder et al. 2017).

It is difficult to compare the present study to the literature, because of differences in design and patient characteristics, e.g., predominance of NSCLC. Barlesi et al. treated NSCLC patients with brain metastases ineligible for (radio)surgery with up to six cycles of cisplatin and pemetrexed every 3 weeks (Barlesi et al. 2011). WBRT was given in case of disease progression or at chemotherapy completion. The primary endpoint was objective response rate (RR) in the brain. Forty-three patients were enrolled. Cerebral, extracerebral, and overall RR by intent to treat analysis were 42%, 35%, and 35%, respectively. Median survival time and time to progression were 7.4 and 4.0 months, respectively. A different phase II study included patients with non-squamous NSCLC and asymptomatic untreated brain metastases who received first-line bevacizumab plus carboplatin and paclitaxel every 3 weeks, or, if already treated earlier, second-line bevacizumab plus erlotinib (Besse et al. 2015). Six-month progression-free survival (PFS) was the primary endpoint. In the first-line cohort (*n* = 67), 6-month PFS rate was 56.5% with a median PFS of 6.7 months and median overall survival of 16.0 months. Investigator-assessed overall RR was 63% (61% in intracranial lesions and 64% in extracranial lesions).

Monnet et al. reported a phase III trial, which randomized NSCLC patients with asymptomatic brain metastases to receive upfront brain radiotherapy (WBRT or other) and subsequent chemotherapy (platin-pemetrexed and bevacizumab in eligible patients, followed by maintenance pemetrexed with or without bevacizumab) or the same chemotherapy with brain radiotherapy only at cerebral progression (Monnet et al. 2021). The primary endpoint was PFS. Unfortunately, the trial was stopped early because of slow recruitment. Among 95 included patients, 91 were randomized (adenocarcinoma: 92%, extracranial metastases: 58%, without differences between arms.) Significantly, more patients in the radiotherapy arm received radiation compared with those in the chemotherapy alone arm (87% versus 20%; *p* < 0.001). There were no significant differences for median PFS: 4.7 versus 4.8 months. Median overall survival was 8.5 and 8.3 months, respectively (close to the present study).

Recently, ICI approval has changed the treatment paradigms. Goldberg et al. performed a phase 2 trial of pembrolizumab in patients with NSCLC (or melanoma) with untreated brain metastases (*n* = 42) (Goldberg et al. 2020). Some patients had brain metastases progressing after previous radiotherapy, but none had neurological symptoms or corticosteroid requirement. Patients were separated in two cohorts: cohort 1 had PD-L1 expression of at least 1% and cohort 2 had PD-L1 less than 1% or unevaluable. The primary endpoint was the proportion of patients achieving a brain metastasis response. Eleven of 37 patients (30%) in cohort 1 had a brain metastasis response. There were no responses in cohort 2. A recent meta-analysis included 12 studies with a total of 566 patients (Teixeira Loiola de Alencar 2021). ICI therapy resulted in an intracranial RR of 16% and a disease control rate of 45%. There was no difference among patients with brain metastases who were treated with radiation before ICI start and those who were treated with ICI only. However, need for radiotherapy, timing, and sequencing are still under debate in the literature (Li and Yu 2020; Scoccianti et al. 2021). The present study did not include a sufficiently large ICI cohort to contribute to the controversial discussion.

A pooled analysis of three KEYNOTE trials included 171 patients with stable baseline brain metastases (Powell et al. 2021). Their median overall survival was 18.8 months with pembrolizumab plus chemotherapy and 7.6 months with chemotherapy, and median PFS was 6.9 months and 4.1 months, respectively. Objective response rates were higher and duration of response longer with pembrolizumab plus chemotherapy versus chemotherapy. These data led the authors to conclude that combined treatment is a standard-of-care treatment option for treatment-naïve patients with NSCLC and stable brain metastases. As mentioned in the previous paragraph on ICI alone, the present study did not include many patients treated with ICI and chemotherapy. Ideally, larger studies in more homogeneous patient populations, comparable to those for targeted agents (Erickson et al. 2020), will be performed in the future.

Despite its limitations, this study revealed interesting insights. Furthermore, it encourages additional validation of established prognostic models and factors for patients with brain metastases from lung cancer, in the setting of primary systemic therapy. On one hand, the fact that the number of brain metastases and age were not associated with survival in our study might be a result of study size and limited statistical power. On the other hand, eligibility for chemotherapy likely leads to a shift in some baseline characteristics (better organ function, less comorbidity, etc.), potentially resulting in diminished applicability of models that were developed in less-restricted groups of patients, typically patients managed with brain irradiation (Sperduto et al. 2017; Nieder et al. 2018b).

## Data Availability

Data will not be shared, but a copy of relevant baseline parameters can be provided to researchers attempting to pool data from several institutions for large-scale analyses.
